# Motivation in medical students: a PhD thesis report

**DOI:** 10.1007/s40037-012-0016-1

**Published:** 2012-06-19

**Authors:** Rashmi Kusurkar

**Affiliations:** 1 Research in Medical Education, Institute for Education and Training, VU University Medical Center, A-114, PO Box 7057, 1007 MB Amsterdam, the Netherlands; 2 Center for Research and Development of Education, University Medical Center, Utrecht, the Netherlands

**Keywords:** Motivation, Medical students, Intrinsic motivation, Autonomy, Competence, Feedback

## Abstract

The aims of this thesis were to gather insights and investigate the factors influencing, outcomes and applications of medical students' motivation. This thesis consists of three literature reviews, four research papers and two application papers. Two research studies investigated the relationships of student motivation with study strategy, effort and academic performance through structural equation modelling and cluster analysis. The relationships of age, maturity, gender and educational background with motivation were investigated through multiple regression analysis. The results of this thesis were 1. Developments in medical education appear to have undervalued student motivation. 2. Motivation is an independent variable in medical education; intrinsic motivation is significantly associated with deep study strategy, high study effort and good academic performance. 3. Motivation is a dependent variable in medical education and is significantly affected by age, maturity, gender, educational background; intrinsic motivation is enhanced by providing students with autonomy, feedback and emotional support. 4. Strength of motivation for medical school can be reliably measured by Strength of Motivation for Medical School questionnaire. The conclusion of this thesis was that it is important to give consideration to motivation in medical education because intrinsic motivation leads to better learning and performance and it can be enhanced through giving students autonomy in learning, feedback about competence and emotional support.

## Introduction

The importance of motivation in learning behavior and performance is not well substantiated in medical education. There is sometimes focus on increasing the quantity of motivation, but the how and why need more evidence. The aims of this thesis were to investigate the outcomes of motivation in medical students and to determine the factors influencing motivation and ways to enhance it. The main research questions were: (1) Is motivation a predictor for learning and academic performance? (2) What factors affect motivation? (3) How can intrinsic motivation be enhanced?

## Methods

The literature was reviewed to explore whether the developments in medical curricula were geared towards student motivation. It was also reviewed to determine how motivation was investigated as a dependent and an independent variable in medical education. The validity of a scale to measure the strength of motivation for medical school was investigated. Two research studies investigated the relationships of student motivation with study strategy, effort and academic performance through structural equation modelling and cluster analysis. Studying motivation using an approach which combined quantity and quality of motivation was proposed through a review of the literature. As a dependent variable the relationships of age, maturity, gender, and educational background with motivation were investigated through multiple regression analysis. Applications of this research were described and recommendations were made.

## Results

1. Developments in medical education appear to have undervalued student motivation.
2. Motivation is both an independent and a dependent variable in medical education.
3. Motivation as an independent variable, particularly intrinsic motivation, is significantly associated with deep study strategy, high study effort, and good academic performance.
4. Motivation as a dependent variable is significantly affected by age, maturity, gender and educational background, and intrinsic motivation is enhanced by providing students autonomy, feedback, and emotional support.
5. Strength of motivation for medical school can be reliably measured by the Strength of Motivation for Medical School questionnaire.

## Discussion

Student motivation has been given low consideration in medical education and medical curricular reforms are not geared towards enhancing student motivation. Studying motivation through an approach giving importance to both the quality and quantity of motivation is recommended. Intrinsic motivation (learning for the sake of learning) leads to better learning and performance as compared with extrinsic motivation (learning for reward), and can be enhanced by providing students with autonomy in learning, feedback on their performance, and emotional support.

## Conclusions

It is important to give consideration to motivation in medical students because intrinsic motivation leads to better learning and performance and it can be enhanced through teaching–learning practices.

